# Refraction and ocular biometric parameters in 3-to 6-year-old preschool children : a large-scale population-based study in Chengdu, China

**DOI:** 10.1186/s12886-024-03467-w

**Published:** 2024-05-06

**Authors:** Jingyu Mu, Zengrui Zhang, Xiaoxiao Wu, Siyu Chen, Haoming Geng, Junguo Duan

**Affiliations:** 1 Chengdu University of TCM, Chengdu, Sichuan China; 2 Eye college of Chengdu University of TCM, Chengdu, Sichuan China; 3 Ineye Hospital of Chengdu University of TCM, Chengdu, Sichuan China; 4 Key Laboratory of Sichuan Province Ophthalmopathy Prevention & Cure and Visual Function Protection with TCM Laboratory, Chengdu, Sichuan China; 5 Binzhou hospital of TCM, Binzhou, Shandong China; 6 Retinal Image Technology and Chronic Vascular Disease Prevention & Control and Collaborative Innovation Center, Chengdu, Sichuan China

**Keywords:** Myopia, Preschool children, Refractive errors, Ocular biometric parameters

## Abstract

**Purpose:**

To understand the ocular biometric parameters characteristics and refractive errors in 3-to 6-year-old preschool children in Chengdu, China, and to investigate the prevalence of refractive errors.

**Method:**

A school-based cross-sectional study was conducted in Chengdu from 2020 to2022 with a total of 666 kindergartens. All children were measured by non-cycloplegic autorefraction and uncorrected visual acuity (UCVA) and ocular biometric parameters. Finally, univariate linear regression models were used to analyze the relationship between ocular biometric parameters and refraction.

**Results:**

A total of 108,578 preschool children aged 3–6 underwent examinations, revealing a myopia prevalence of 6.1%. The mean axial length (AL), keratometry (K), corneal radius (CR), axial length/corneal radius (AL/CR) Ratio, central corneal thickness (CCT), anterior chamber depth (ACD), lens thickness (LT), and vitreous chamber depth (VCD) were 22.35 ± 0.69 mm, 43.35 ± 1.58 D, 7.80 ± 0.28 mm, 2.87 ± 0.08, 533.31 ± 32.51 μm, 2.70 ± 0.28 mm, 3.91 ± 0.27 mm, and 15.20 ± 0.68 mm, respectively. With increasing age, AL, CR, AL/CR ratio, CCT, ACD, LT, and VCD also increased. Regardless of age, males consistently exhibited longer AL, flatter corneal curvature, shallower ACD, thicker CCT, thinner LT, and longer VCD compared to females. AL, K, CR, LT, and VCD all showed significant linear relationships with SE (all *P* < 0.001) in univariate linear regression analysis after adjusting for gender and age.

**Conclusion:**

The prevalence of myopia among preschool children aged 3–6 in Chengdu is relatively low. Ocular biometric parameters affecting refractive errors include AL, K, CR, LT, and VCD. The preschool period serves as a critical phase for myopia prevention and control.

## Introduction

The rapid increase in the prevalence of myopia and the associated risks of high myopia complications have become a focal societal concern. The human eye is a complex optical system closely interconnected with components such as the tear film, cornea, anterior chamber depth, lens, vitreous, and other refractive elements. Throughout the normal process of ocular growth, the delicate balance between refractive components, including the cornea and lens, and AL, ultimately determines the refractive status of the eye [1–4]. In 2020, the Chinese National Health Commission released a myopia prevalence of 52.7% among children and adolescents in China. This rate was significantly higher than that in the Americas, Europe, Africa and other economically disadvantaged regions [5].According to estimates, approximately 49.8% of the global population was projected to be myopic, with 9.8% of individuals experiencing high myopia by the year 2050 [6].The progression of myopia to high myopia was prone to cause complications, including retinal fissures, detachment, and choroidal neovascularization. These complications can lead to permanent visual damage, making myopia a major contributing factor to visual impairment and blindness [7].

In recent years, myopia has shown a trend of high incidence and onset at younger ages in children and adolescents. Experts and scholars believed that the critical point for myopia prevention and control should be extended to include preschool children. They emphasize the importance of early intervention, including examinations to assess hyperopia reserve. Additionally, objective evaluation of refractive status can be achieved through ocular biometric parameters such as axial length (AL), axial length/corneal radius (AL/CR) Ratio, and lens power (LP) [8–11]. Therefore, the prevention and control of myopia are urgent, especially when considering preschool children who are in a sensitive period of visual development.

Currently, cycloplegic refraction is internationally recognized as the gold standard for diagnosing myopia [12]. However, due to its time-consuming nature and potential short-term side effects such as myopic blur and photophobia, as well as children’s resistance to eye drops and parental concerns about potential side effects, it is not recommended as the preferred method for large-scale myopia screening in children and adolescents. Uncorrected visual acuity (UCVA) testing is widely used worldwide for screening myopia in children and adolescents, although UCVA testing is simple and rapid, it cannot differentiate between refractive errors [13]. Additionally, non-cycloplegic refraction can lead to overestimation of myopia prevalence [14]. Therefore, the Chinese National Health Commission recommends the rapid and convenient methods such as UCVA testing and non-cycloplegic autorefraction for screening potential myopia in children and adolescents. Thus, this study will also adopt non-cycloplegic autorefraction and UCVA together to define myopia.

However, there was limited data on ocular biometric parameters and refractive errors in preschool children. Chengdu, located in the southwestern part of China, is one of the largest and most significant cities in the region. This study focuses on preschool children aged 3–6 in Chengdu. By analyzing the relationship between ocular biometric parameters and refractive errors, the research aims to further elucidate the patterns of ocular growth, and refractive status changes in preschool children. Additionally, the study seeks to compare differences among parameters such as age and gender. Finally, the evaluation of ocular biometric parameters in preschool children aged 3–6 aims to assess their role in monitoring refractive errors. The hope is that this research will contribute to the efforts in myopia prevention and provide assistance in this regard.

## Methods

### Study design and population

This study was a population-based cross-sectional research conducted in kindergartens in Chengdu from 2020 to 2022. We employed a stratified random cluster sampling method and selected a total of 666 kindergartens, involving 108,578 participants. Inclusion criteria comprised: (1) preschool students enrolled in kindergartens in Chengdu; (2) aged between 3 and 6 years. Exclusion criteria encompassed: (1) patients with various types of glaucoma, corneal diseases, lens disorders, retinal diseases, optic nerve diseases, etc.; (2) participants with amblyopia, strabismus, significant anisometropia, or severe visual functional impairment; (3) participants with entropion, severe conjunctivitis, and the like; (4) participants with poor compliance, mental illness, or cognitive disorders. This study received approval from the Ethics Committee of Chengdu University of Traditional Chinese Medicine Ineye Hospital(2019yh-007). All research methods adhered to the principles outlined in the “Declaration of Helsinki”. Prior to conducting the study, the objectives and methods were presented to the principals, teachers, and parents of the participating schools to obtain informed consent, and signatures were obtained accordingly.

### Eye examination

With the assistance of the Chengdu Education Bureau and Health Bureau, Chengdu Traditional Chinese Medicine University Ineye Hospital collected student information in advance. This information included school type, school name, grade, class, name, gender, age, ID number, and guardian’s phone number. An eye health record was established through the Eye Health Records System, containing all student information and a unique identification code. The examination results were transmitted using this identification code.

The research team consisted entirely of ophthalmologists, optometrists, and ophthalmic nurses, all of whom have undergone standardized training. All study subjects underwent ophthalmic examinations, including uncorrected visual acuity (UCVA), corrected visual acuity with glasses, non-cycloplegic autorefraction, and ocular biometric parameter assessments. Each student underwent an uncorrected visual acuity test using the international standard visual acuity chart for the E letter (GB11533-2011). If glasses were worn, visual acuity with glasses was also assessed. The autorefraction (model RM-800; Topcon, Tokyo, Japan) was used to measure non-cycloplegic autorefraction. Three measurements were taken for each eye, and the spherical difference between any two measurements was required to be < 0.5 D; otherwise, additional measurements were performed, and the average of valid measurements was recorded as the final result. Ocular biometric parameters were conducted using the SUOER Ophthalmic Optical Biometer (SW-9000, Tianjin Shisuowei Electronic Technology Co., Ltd). Each eye underwent three measurements, with the instrument automatically checking the quality of each measurement. If a measurement was deemed inadequate, additional measurements were taken. The average of the three test results was calculated and recorded as the outcome.

### Definitions

The prevalence of refractive errors was determined using spherical equivalent (SE) based on non-cycloplegic autorefraction and uncorrected visual acuity (UCVA) values [15]. The following definitions and classifications were employed: Myopia was defined as non-cycloplegic SE ≤ -0.50 D + UCVA > 0.3 log MAR (age 3), > 0.2 log MAR (ages 4–5), > 0 log MAR (age ≥ 6) [16, 17]. Hyperopia: SE ≥ + 0.50 D; values below this threshold were considered emmetropia.

### Statistical analysis

The data processing and analysis were conducted using SPSS software (version 26.0, Chicago, IL, USA). Continuous variables that follow a normal distribution were described as $\bar x \pm s$, while those that do not follow a normal distribution were described as M (P25, P75). Independent sample t-tests were used for comparisons between two groups, and one-way analysis of variance (ANOVA) was used for multiple group comparisons. Categorical variables were presented as n (%), and differences between two groups (or multiple groups) were assessed using the chi-square test (or Fisher’s exact test when conditions for R×C were not met). For the comparison of ordinal data among multiple groups, the Kruskal-Wallis H test was employed. In cases where the normality assumption was not met, the Mann-Whitney test was used for comparisons. Univariate linear regression analysis was performed to analyze the correlation between spherical equivalent (SE) and age, gender, and ocular biometric parameters. The significance level was set at α = 0.05. Given the high correlation (*r* = 0.88) between the SE of the left and right eyes, the analysis in this study focused on the right eye.

## Results

A total of 108,578 children participated in this study, with 666 kindergartens ultimately completing the examinations for uncorrected visual acuity (UCVA), corrected visual acuity with glasses, non-cycloplegic autorefraction, and ocular biometric parameters. Among them, 55,326 were boys (51.0%), and 53,252 were girls (49.0%). Additionally, there were 19,421, 35,614, 37,115, and 16,428 participants aged 3, 4, 5, and 6 years, respectively. The average age was 4.47 ± 0.953 years.

The M (P25, P75) of spherical equivalent (SE) for preschool children aged 3–6 was 0.25(0,0.625) D in Chengdu. The SE respectively was 0.25(-0.125,0.625) D, 0.25(0,0.625) D, 0.25(0,0.625) D, and 0.25(-0.125,0.5) D, at ages 3, 4, 5, and 6. Overall SE and SE stratified by gender showed statistically significant differences (all *P* < 0.01). Additionally, there was a statistically significant difference in SE between genders in the overall group and at age 5 (all *P* < 0.05). However, no statistically significant differences in SE between genders were observed at ages 3, 4, and 6 (all *P* > 0.05) (Table 1).

**Table 1:** Refractive and ocular biometric characteristics in 3-to 6-year-old preschool children

Characteristic	3y	4y	5y	6y	total	*P*
SE						
Female	0.25(-0.125,0.625)	0.375(0,0.625)	0.25(0,0.625)	0.25(-0.125,0.625)	0.25(0,0.625)	< 0.001
Male	0.25(-0.125,0.625)	0.25(-0.125,0.625)	0.25(0,0.5)	0.25(-0.125,0.5)	0.25(-0.125,0.625)	< 0.001
Total	0.25(-0.125,0.625)	0.25(0,0.625)	0.25(0,0.625)	0.25(-0.125,0.5)	0.25(0,0.625)	0.002
P	0.061	0.063	0.017	0.045	< 0.001	
AL						
Female	21.76 ± 0.58	21.99 ± 0.59	22.22 ± 0.62	22.38 ± 0.63	22.09 ± 0.64	< 0.001
Male	22.26 ± 0.59	22.51 ± 0.61	22.74 ± 0.63	22.91 ± 0.63	22.60 ± 0.65	< 0.001
Total	22.01 ± 0.63	22.25 ± 0.65	22.49 ± 0.67	22.65 ± 0.68	22.35 ± 0.69	< 0.001
P	< 0.001	< 0.001	< 0.001	< 0.001	< 0.001	
K						
Female	43.80 ± 1.53	43.76 ± 1.52	43.67 ± 1.53	43.69 ± 1.58	43.73 ± 1.53	< 0.001
Male	43.07 ± 1.50	43.02 ± 1.53	42.95 ± 1.53	42.96 ± 1.55	42.99 ± 1.53	< 0.001
Total	43.43 ± 1.56	43.38 ± 1.57	43.30 ± 1.57	43.31 ± 1.61	43.35 ± 1.58	< 0.001
P	< 0.001	< 0.001	< 0.001	< 0.001	< 0.001	
CR						
Female	7.71 ± 0.27	7.72 ± 0.27	7.74 ± 0.27	7.74 ± 0.28	7.73 ± 0.27	< 0.001
Male	7.85 ± 0.28	7.86 ± 0.28	7.87 ± 0.28	7.87 ± 0.28	7.86 ± 0.28	< 0.001
Total	7.78 ± 0.27	7.79 ± 0.28	7.805 ± 0.29	7.804 ± 0.28	7.80 ± 0.28	< 0.001
P	< 0.001	< 0.001	< 0.001	< 0.001	< 0.001	
AL/CR ratio						
Female	2.82 ± 0.08	2.85 ± 0.08	2.87 ± 0.08	2.89 ± 0.08	2.86 ± 0.08	< 0.001
Male	2.84 ± 0.08	2.87 ± 0.08	2.89 ± 0.08	2.91 ± 0.09	2.88 ± 0.08	< 0.001
Total	2.83 ± 0.08	2.86 ± 0.08	2.88 ± 0.08	2.90 ± 0.08	2.87 ± 0.08	< 0.001
P	< 0.001	< 0.001	< 0.001	< 0.001	< 0.001	
CCT						
Female	529.04 ± 32.66	530.07 ± 31.65	532.28 ± 31.70	534.67 ± 31.68	531.32 ± 31.91	< 0.001
Male	532.97 ± 33.98	533.39 ± 32.47	536.53 ± 32.62	538.74 ± 33.10	535.22 ± 32.96	< 0.001
Total	531.02 ± 33.39	531.75 ± 32.11	534.45 ± 32.24	536.79 ± 32.49	533.31 ± 32.51	< 0.001
P	< 0.001	< 0.001	< 0.001	< 0.001	< 0.001	
ACD						
Female	2.55 ± 0.27	2.61 ± 0.26	2.68 ± 0.26	2.74 ± 0.26	2.64 ± 0.27	< 0.001
Male	2.67 ± 0.28	2.73 ± 0.27	2.79 ± 0.27	2.86 ± 0.27	2.76 ± 0.27	< 0.001
Total	2.61 ± 0.28	2.67 ± 0.27	2.73 ± 0.27	2.80 ± 0.27	2.70 ± 0.28	< 0.001
P	< 0.001	< 0.001	< 0.001	< 0.001	< 0.001	
LT						
Female	4.04 ± 0.28	3.97 ± 0.26	3.89 ± 0.26	3.83 ± 0.25	3.93 ± 0.27	< 0.001
Male	4.00 ± 0.28	3.93 ± 0.27	3.85 ± 0.26	3.79 ± 0.25	3.89 ± 0.28	< 0.001
Total	4.02 ± 0.28	3.95 ± 0.27	3.87 ± 0.26	3.81 ± 0.25	3.91 ± 0.27	< 0.001
P	< 0.001	< 0.001	< 0.001	< 0.001	< 0.001	
VCD						
Female	14.64 ± 0.58	14.88 ± 0.59	15.12 ± 0.61	15.28 ± 0.63	14.98 ± 0.64	< 0.001
Male	15.06 ± 0.59	15.32 ± 0.61	15.56 ± 0.63	15.72 ± 0.63	15.42 ± 0.65	< 0.001
Total	14.85 ± 0.62	15.10 ± 0.64	15.34 ± 0.66	15.51 ± 0.67	15.20 ± 0.68	< 0.001
P	< 0.001	< 0.001	< 0.001	< 0.001	< 0.001	

**Fig. 1:**
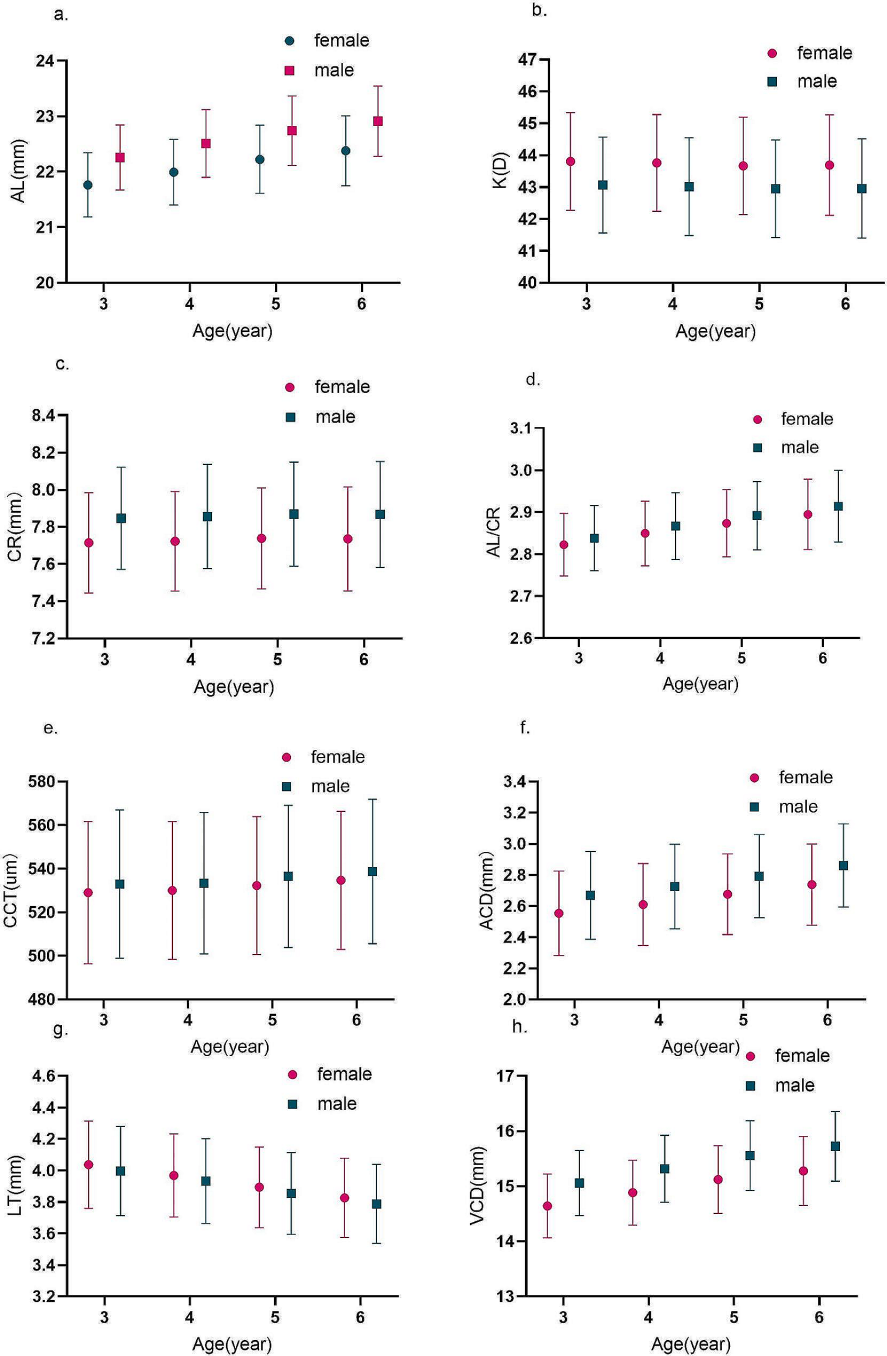
Trends in the ocular biometric parameters between female and male among different age groups. **a**. Trends in the AL among different age groups; **b**. Trends in the K among different age groups; **c**. Trends in the CR among different age groups; **d**. Trends in the AL/CR ratio among different age groups; **e**. Trends in the CCT among different age groups; **f**. Trends in the ACD among different age groups; **g**. Trends in the LT among different age groups; **h**. Trends in the VCD among different age groups

The age and gender distribution of ocular biometric parameters was presented in Table 1; Fig. 1. The average for AL, CR, AL/CR ratio, CCT, ACD, and VCD were 22.35 ± 0.69 mm, 7.80 ± 0.28 mm, 2.87 ± 0.08, 533.31 ± 32.51 μm, 2.70 ± 0.28 mm, and 15.20 ± 0.68 mm, respectively. Boys had longer AL, CR, AL/CR ratio, CCT, ACD, and VCD compared to girls, and these differences were statistically significant in all age groups (all *P* < 0.001). With increasing age, both overall and gender-stratified for AL, CR, AL/CR ratio, CCT, ACD, and VCD showed a general increasing trend (all *P* < 0.001), with no statistically significant differences in CR at ages 5 and 6, in CCT at ages 3 and 4 (all *P* > 0.05). The average of K and LT were 43.35 ± 1.58 D and 3.91 ± 0.27 mm, respectively. In all age groups, boys had smaller K and LT compared to girls, indicating that boys had flatter corneal curvature, and these differences were statistically significant (all *P* < 0.001). With increasing age, both overall and gender-stratified K and LT generally showed a decreasing trend (all *P* < 0.001), with no statistically significant differences in K at ages 3 and 4, 5 and 6 (*P* > 0.05).

**Fig. 2:**
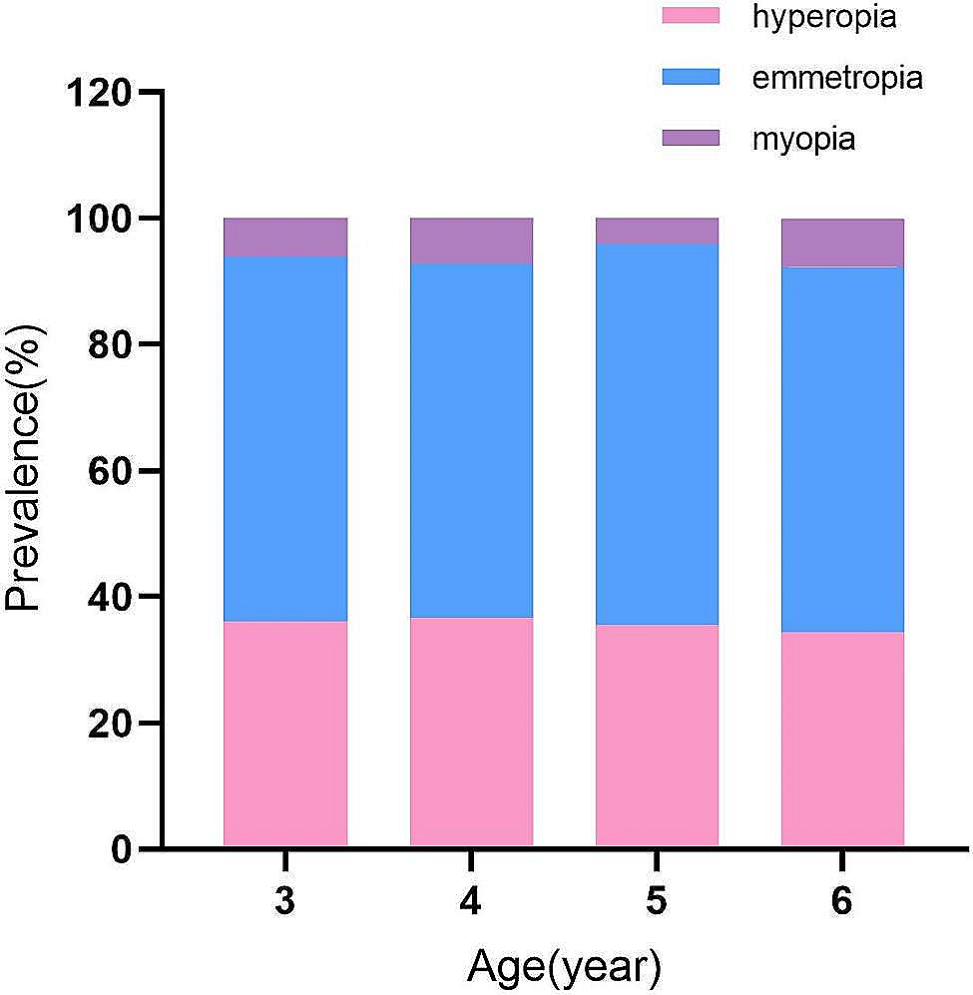
Bar graph showing distributions of the prevalence of refractive error among different age groups

The distribution of refractive errors across different age groups was presented in Table 2; Fig. 2. A total of 6,619 children (6.1%) were diagnosed with myopia, with a myopia prevalence of 6.1% (1,177/19,421) at 3 years old, 7.4% (2,628/35,614) at 4 years old, 4.2% (1,542/37,115) at 5 years old, and 7.7% (1,272/16,428) at 6 years old, and the myopia prevalence differed significantly across various age groups (*P* < 0.001). However, there were no statistically significant differences in myopia prevalence between genders in the overall children and within each age group (all *P* > 0.05). On the other hand, 38,846 children (35.8%) were diagnosed with hyperopia and the prevalence of hyperopia varied significantly across different age groups (*P* < 0.001). A total of 63,113 children (58.1%) were emmetropic, and the prevalence of emmetropia differed significantly across various age groups (*P* < 0.001).

**Table 2:** Distribution of prevalence of refractive errors in 3-to 6-year-old preschool children

Characteristic	3y	4y	5y	6y	total	*P*
N	19,421	35,614	37,115	16,428	108,578	
Hyperopia						
Total	7016(36.1)	13,019(36.6)	13,172(35.5)	5639(34.3)	38,846(35.8)	< 0.001
Female	3507(36.4)	6602(37.6)	6517(35.9)	2802(35.6)	19,428(36.5)	0.002
Male	3509(35.9)	6417(35.5)	6655(35.1)	2837(33.2)	19,418(35.1)	< 0.001
P	0.485	< 0.001	0.15	0.001	< 0.001	
Emmetropia						
Total	11,228(57.8)	19,967(56.1)	22,401(60.4)	9517(57.9)	63,113(58.1)	< 0.001
Female	5565(57.7)	9683(55.1)	10,898(60)	4458(56.6)	30,604(57.5)	< 0.001
Male	5663(57.9)	10,284(57)	11,503(60.7)	5059(59.1)	32,509(58.8)	< 0.001
P	0.772	0.001	0.125	0.001	< 0.001	
Myopia						
Total	1177(6.1)	2628(7.4)	1542(4.2)	1272(7.7)	6619(6.1)	< 0.001
Female	571(5.9)	1273(7.3)	761(4.2)	615(7.8)	3220(6.0)	< 0.001
Male	606(6.2)	1355(7.5)	781(4.1)	657(7.7)	3399(6.1)	< 0.001
P	0.42	0.359	0.761	0.759	0.505	

The distribution of ocular biometric parameters across different refractive status was presented in Table 3. The average of AL with hyperopia, emmetropia, and myopia were 22.26 ± 0.70 mm, 22.39 ± 0.67 mm, and 22.47 ± 0.80 mm, respectively, and there was a statistically significant difference in AL (*P* < 0.001).The average of K with hyperopia, emmetropia and myopia were 43.32 ± 1.55 D, 43.36 ± 1.59 D, and 43.47 ± 1.61 D, there was a statistically significant difference in K (*P* < 0.001). The average of AL/CR ratio with hyperopia, emmetropia and myopia were 2.86 ± 0.08, 2.87 ± 0.08, and 2.89 ± 0.10, and the differences in AL/CR ratio were statistically significant (*P* < 0.001). The average of CCT with hyperopia, emmetropia, and myopia were 533.18 ± 32.74 μm, 533.39 ± 32.28 μm and 533.21 ± 33.28 μm, and there was no statistically significant difference in CCT (*P* > 0.05). The average of ACD with hyperopia, emmetropia and myopia were 2.70 ± 0.28 mm, 2.70 ± 0.28 mm and 2.71 ± 0.30 mm, and there was no statistically significant difference in ACD between the hyperopia and the emmetropia group (*P* > 0.05), while the myopia group had statistical significance compared to the hyperopia and emmetropia groups (*P* < 0.001). The average of LT with hyperopia, emmetropia and myopia were 3.89 ± 0.27 mm, 3.92 ± 0.27 mm, and 3.95 ± 0.30 mm, and the difference in LT was statistically significant (*P* < 0.001). The average of VCD with hyperopia, emmetropia and myopia was 15.14 ± 0.68 mm, 15.23 ± 0.66 mm, and 15.27 ± 0.80 mm, and the difference in VCD was statistically significant (*P* < 0.001).

**Table 3:** The relationship between refractive errors and ocular biometric parameters

Characteristic	hyperopia	emmetropia	myopia	total	*P*
N	38,846	63,113	6619	108,578	< 0.001
SE	0.625(0.5,0.875)	0.125(-0.125,0.25)	-1.0(-1.5,-0.625)	0.25(0,0.625)	< 0.001
AL	22.26 ± 0.70	22.39 ± 0.67	22.47 ± 0.80	22.35 ± 0.69	< 0.001
K	43.32 ± 1.55	43.36 ± 1.59	43.47 ± 1.61	43.35 ± 1.58	< 0.001
CR	7.80 ± 0.28	7.79 ± 0.28	7.78 ± 0.29	7.80 ± 0.28	< 0.001
AL/CR	2.86 ± 0.08	2.87 ± 0.08	2.89 ± 0.10	2.87 ± 0.08	< 0.001
CCT	533.18 ± 32.74	533.39 ± 32.28	533.21 ± 33.28	533.31 ± 32.51	0.585
ACD	2.70 ± 0.28	2.70 ± 0.28	2.71 ± 0.30	2.70 ± 0.28	0.001
LT	3.89 ± 0.27	3.92 ± 0.27	3.95 ± 0.30	3.91 ± 0.27	< 0.001
VCD	15.14 ± 0.68	15.23 ± 0.66	15.27 ± 0.80	15.20 ± 0.68	< 0.001

**Fig. 3:**
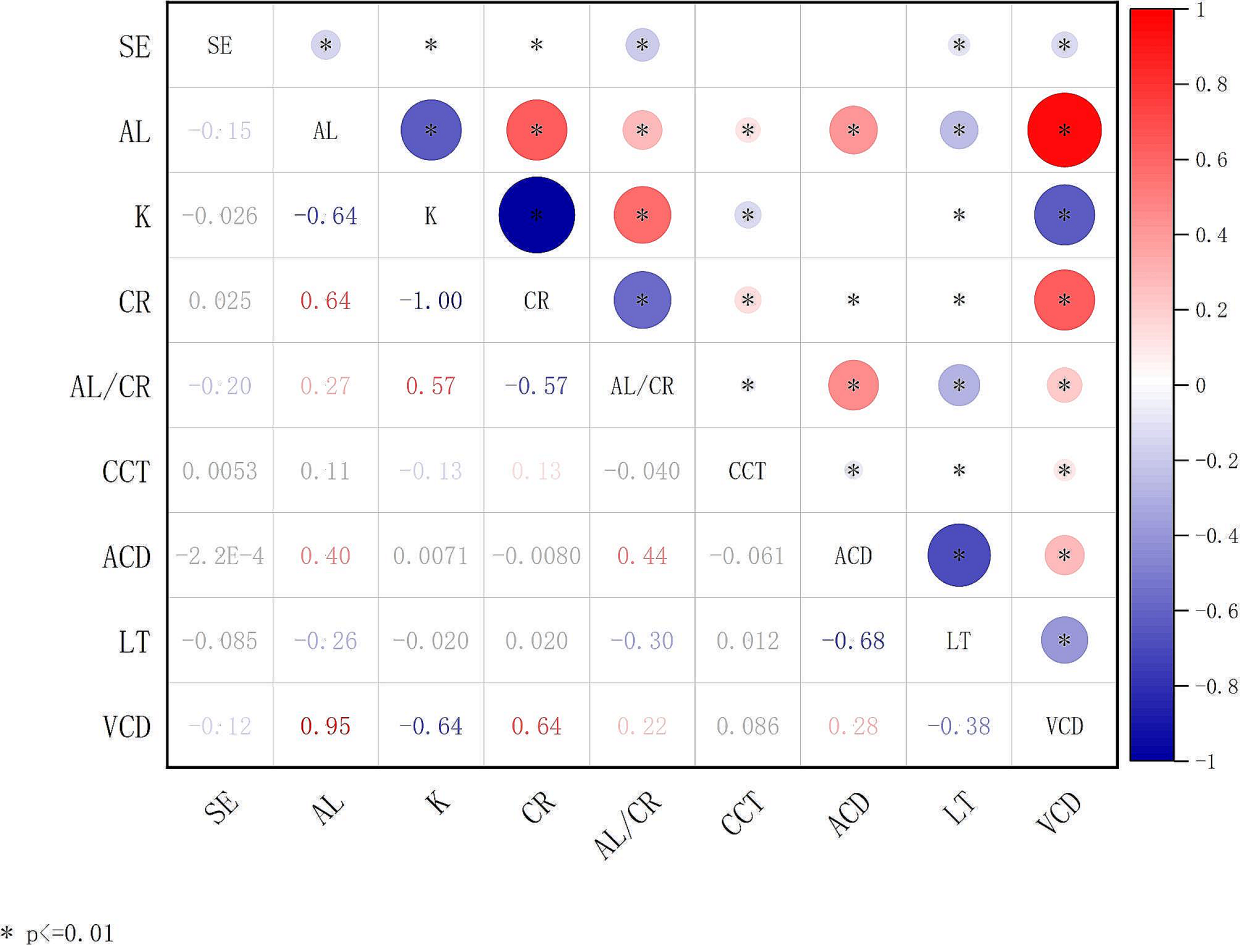
The correlation between SE and ocular biometric parameters

Pearson correlation analysis revealed that SE was positive correlated with CR (*r* = 0.025, *P* < 0.001). On the other hand, SE showed negatively correlation with AL, K, AL/CR ratio, LT and VCD (*r* = -0.153, -0.026,-0.195,-0.085 and − 0.122, all *P* < 0.001). There was no correlation between SE and CCT, ACD (*P* > 0.05)(Fig. 3).

Table 4 summarizes the results of the univariate linear regression models for ocular biometric parameters and SE. After adjusting for gender and age, AL, K, CR, LT, and VCD all showed significant linear relationships with SE (β = -0.226, -0.015, 0.083, -0.258, -0.178; all *P* < 0.001), while there were no significant linear relationships between CCT and ACD with SE (all *P* > 0.05).

**Table 4:** Linear regression coefficients with 95% confidence interval for SE and ocular biometry (control for age and gender)

Parameters	β	95%CI	*P* value
**AL**	-0.226	-0.234 to -0.219	< 0.001
**K**	-0.015	-0.018 to -0.012	< 0.001
**CR**	0.083	0.065 to 0.100	< 0.001
**CCT**	0.000	0.000 to 0.000	0.082
**ACD**	-0.002	-0.019 to 0.016	0.865
**LT**	-0.258	-0.276 to -0.241	< 0.001
**VCD**	-0.178	-0.185 to -0.170	< 0.001

Age and gender were controlled in the analysis

SE: spherical equivalent; AL: axial length; K: keratometry; CR: corneal curvature radius; CCT: center cornea thickness; ACD: anterior chamber depth; LT: lens thickness; VCD: vitreous chamber depth;

## Discussion

This study investigated the distribution and developmental patterns of ocular biometric parameters and refractive status in preschool children with respect to age and gender in Chengdu, China. The results of the study indicated that the prevalence of myopia was relatively low among preschool children aged 3–6 in Chengdu (6.1%). Furthermore, as age increases, the prevalence of myopia remained relatively stable. With advancing age, children exhibit varying degrees of changes in ocular biometric parameters such as AL, CR, ACD, CCT, LT, and VCD, suggesting a close association between these parameters and the developmental processes of children’s eyes. Additionally, the study identified gender differences in these ocular biometric parameters, with boys showing significant variations from girls in certain parameters. This was crucial for understanding the normal developmental processes of the eyes in preschool children and the impact of gender on ocular biometric parameters.

In this study, the prevalence of myopia among preschool children aged 3–6 in Chengdu was 6.1% (non-cycloplegia), similar to the myopia prevalence among children aged 4–6 in Shanghai, China, which was 5.9% (non-cycloplegia) [18]. However, the prevalence of myopia among children aged 3–6 in Beijing was 1.93% (post-cycloplegia) [19], it was 1.3% in Shenzhen(post-cycloplegia) [20], and it was 3.7% in Shanghai(post-cycloplegia) [21]. Additionally, the myopia prevalence among non-Hispanic White and Asian children aged 0.5-6 years was 1.20% and 3.98% (post-cycloplegia) [22], which were significantly lower than the findings of our study. A study involving 6-year-old children from Hong Kong reported a myopia prevalence of 17.6% (non-cycloplegic) [23]. The above-mentioned studies collectively suggest a relatively low prevalence of myopia among preschool children. It also indicates that the myopia prevalence may be influenced by factors such as measurement methods (cycloplegia, etc.), diagnostic criteria, geographical location, and ethnic/racial differences.

Furthermore, research indicated that the prevalence of myopia in 5-year-old children was significantly lower compared to those aged 3, 4, and 6 years, and it has also been verified in a number of studies in Chengdu, Guangzhou, and other places. In a study involving children aged 3 to 6 in Chengdu, results from non-cycloplegic refraction screening indicated a lower prevalence of myopia among 5-year-olds compared to other age groups [16]. Similarly, a cross-sectional study using cycloplegic refraction in Guangzhou demonstrated comparable findings [24]. However, some studies have shown a contrary trend, with two cycloplegic refraction studies in Shanghai showing that the prevalence of myopia increases with age in preschool children aged 3 to 6 years [21, 25]. Additionally, a cycloplegic refraction study in Beijing revealed an increase in myopia prevalence among children aged 4 to 6 with increasing age [19]. Overall, while the prevalence of myopia among preschool children aged 3 to 6 is generally low and relatively stable, differences in study outcomes across different locations may be influenced by sample size and study methodological factors such as cycloplegia.

The results of this study indicated that the prevalence of hyperopia among children aged 3–6 in Chengdu was 35.8% (non-cycloplegic), which was significantly lower compared to the hyperopia prevalence in Beijing and Shenzhen (cycloplegic, 85.1%, 93.4%) [19]. The substantial difference in hyperopia prevalence between this study and others may be related to the non-cycloplegic refraction. In recent years, many scholars have emphasized the significance of hyperopic reserve in controlling myopia in children. They proposed that the increased duration of near-distance visual activities in children prematurely depletes their physiological hyperopia, accelerated the “emmetropization” process, led to insufficient hyperopic reserve, and resulted in the early onset of myopia [26]. A 2-year longitudinal study by Zadnik et al. [27] on 4,512 children aged 6–11 in the United States, evaluating 13 myopia risk factors, ultimately found an increased risk of myopia in children with hyperopia less than + 0.75 D. This prediction allowed clinicians and scientists to assess the risk of myopia in children using simple and practical measures.

This study indicated that in preschool children aged 3–6, older children tend to have longer AL, generally longer CR, deeper ACD, thicker CCT, thinner LT, and VCD. The growth and development patterns of these ocular biometric parameters were consistent with previous literature [19, 20]. The results aligned with the characteristic of a relatively rapid increase in AL during the preschool period of 3–6 years, similar to findings in other studies [17]. This was primarily due to this stage being a critical period for eye development, where various components of the eye were undergoing rapid growth and development, including the gradual increase in AL. Studies have indicated a negative correlation between AL and K [28]. In the progression of myopia within a certain range of AL growth, the cornea can compensate for the effects of AL growth on myopia through changes in curvature, preventing further progression of myopia [29]. A prospective cohort study [30]found that in both the overall group and the sustained myopia group, the growth and development of the eyeball showed continuous increases in AL, consistent with the findings in this study for 3–6 years, and a continued thinning of LT from 3 to 11 years, followed by an increase in LT after 11 years. This suggested age-related differences in the growth patterns of the lens.

The AL/CR ratio was the ratio of the axial length to the average corneal radius, which served as a parameter for assessing the risk and progression patterns of myopia. The AL/CR ratio greater than 3 was ultimately considered a high-risk indicator for the progression from emmetropia to myopia [31, 32]. According to existing literature, studies conducted in locations such as Shanghai, Shenzhen, Nanjing, and Anyang, Henan, have consistently observed a positive correlation between AL/CR ratio and age, with males exhibiting higher ratios than females. This trend was observed across all age groups studied [33–37], aligning with the findings of this study. This study indicated that in preschool children aged 3–6 years, the AL/CR ratio increased with age, rising from 2.83 ± 0.08 to 2.90 ± 0.08. These values closely resembled the results obtained in studies of preschool children aged 3–6 years in Beijing and Shenzhen [19, 20]. Some scholars have noted that using AL/CR ratio alone for predicting myopia in the 3–5 age group may be relatively inaccurate. For preschool children, it is recommended to use AL/CR ratio or a combination of AL and non-cycloplegic autorefraction to enhance accuracy [38].

ACD was a crucial component of AL. In this study, ACD deepened with increasing age (*P* < 0.001), consistent with findings from other studies [39, 40]. The data also indicated that eyes with longer AL (indicating higher myopia severity) tend to have deeper ACD and thinner LT. In myopic patients, the increased depth of ACD may result from geometric scaling during the process of AL growth [41].As age increases, VCD elongates (*P* < 0.001), with males exhibiting greater VCD than females (*P* < 0.001), aligning with established patterns of ocular growth and development [42, 43]. This was likely a consequence of the continuous elongation of the eye axis, where the elongation of the vitreous chamber was a major contributor, and AL showed a strong correlation with VCD [44, 45].

Furthermore, this study observed gender differences in ocular biometric parameters among preschool children. Typically, males showed significantly larger AL, CR, AL/CR ratio, CCT, ACD, and VCD compared to females, while females exhibited larger K and LT than males. Similar gender differences in ocular parameters have been reported in epidemiological data from countries such as Japan [46], Singapore [47], Germany [48], supporting the consistency of this observation.

This study observed a thickening trend in CCT with increasing age, while there was no significant difference in CCT changes among refractive groups. Some researchers have reported a positive correlation between CCT and SE [49], while others have found thinner CCT in individuals with myopia [50]. Other studies, consistent with the results of this research, concluded that there was no correlation between CCT and SE [51–53]. Additionally, Zhou et al. [54] discovered a negative correlation between CCT and the progression rate of myopia (or SE) and AL elongation rate. Children with thinner CCT exhibited a faster rate of myopia progression and AL growth. Thus, CCT appeared to be a potential risk factor for myopia.

This study had several strengths. Firstly, the study included a large sample size comprising exclusively preschool children from Chengdu. Secondly, there was a relative scarcity of reports on ocular biometric parameters and refractive errors in preschool children aged 3–6 years both domestically and internationally. This study helped fill this gap in the literature. However, there were some limitations to consider. Firstly, due to constraints in time and location, we did not employ cycloplegic autorefraction, which could compromise the accuracy of refractive error measurements. As preschool children were in a developmental stage with strong accommodative ability, uncorrected refractive error can lead to an increase in myopia and a decrease in hyperopia after ciliary muscle paralysis. Therefore, non-cycloplegic refractive error measurements may result in an underestimation of hyperopia. Secondly, this study was cross-sectional, and as such, it cannot assess the changes in refractive parameters before the onset of myopia in preschool children. In future research, we plan to strengthen longitudinal cohort studies to dynamically track changes in refractive parameters, providing a more accurate and scientific understanding of high-risk populations for myopia. This will contribute to the development of effective scientific foundations for preventing and controlling the occurrence and progression of myopia.

## Conclusions

In summary, our study provided clear data on refractive and ocular biometric characteristics, as well as the prevalence of refractive errors, in preschool children aged 3–6 years in Chengdu, China. The prevalence of myopia in preschool children in Chengdu was relatively low. Ocular biometric parameters affecting refractive errors included AL, K, CR, LT and VCD. Among preschool children aged 3–6 years, older children tended to have longer AL, generally longer CR, deeper ACD, thicker CCT, LT, and longer VCD. Additionally, gender differences exist in ocular biometric parameters in this age group.

## Data Availability

No datasets were generated or analysed during the current study.

## References

[1] Scheiman M, Gwiazda J, Zhang Q, Deng L, Fern K, Manny RE, Weissberg E, Hyman L (2016). Longitudinal changes in corneal curvature and its relationship to axial length in the correction of myopia evaluation trial (COMET) cohort. J Optometry.

[2] Ip JM, Huynh SC, Kifley A, Rose KA, Morgan IG, Varma R, Mitchell P (2007). Variation of the contribution from axial length and other oculometric parameters to refraction by age and ethnicity. Invest Ophthalmol Vis Sci.

[3] Mutti DO, Mitchell GL, Jones LA, Friedman NE, Frane SL, Lin WK, Moeschberger ML, Zadnik K (2005). Axial growth and changes in lenticular and corneal power during emmetropization in infants. Invest Ophthalmol Vis Sci.

[4] Iribarren R (2015). Crystalline lens and refractive development. Prog Retin Eye Res.

[5] Grzybowski A, Kanclerz P, Tsubota K, Lanca C, Saw SM (2020). A review on the epidemiology of myopia in school children worldwide. BMC Ophthalmol.

[6] Holden BA, Fricke TR, Wilson DA, Jong M, Naidoo KS, Sankaridurg P, Wong TY, Naduvilath TJ, Resnikoff S (2016). Global prevalence of myopia and high myopia and temporal trends from 2000 through 2050. Ophthalmology.

[7] Wu PC, Huang HM, Yu HJ, Fang PC, Chen CT (2016). Epidemiology of myopia. Asia-Pacific J Ophthalmol (Philadelphia Pa).

[8] Xiang F, He M, Morgan IG (2012). Annual changes in refractive errors and ocular components before and after the onset of myopia in Chinese children. Ophthalmology.

[9] Mu J, Zeng D, Fan J, Liu M, Zhong H, Shuai X, Zhang S (2022). The accuracy of the axial length and axial length/corneal radius ratio for myopia assessment among Chinese children. Front Pead.

[10] Jong M, Sankaridurg P, Naduvilath TJ, Li W, He M (2018). The relationship between Progression in Axial Length/Corneal radius of curvature ratio and spherical equivalent refractive error in myopia. Optometry Vis Science: Official Publication Am Acad Optometry.

[11] Rozema J, Dankert S, Iribarren R, Lanca C, Saw SM (2019). Axial growth and Lens Power Loss at Myopia Onset in Singaporean Children. Invest Ophthalmol Vis Sci.

[12] Flitcroft DI, He M, Jonas JB, Jong M, Naidoo K, Ohno-Matsui K, Rahi J, Resnikoff S, Vitale S, Yannuzzi L (2019). IMI - defining and classifying myopia: a proposed set of standards for clinical and epidemiologic studies. Invest Ophthalmol Vis Sci.

[13] Leone JF, Mitchell P, Morgan IG, Kifley A, Rose KA (2010). Use of visual acuity to screen for significant refractive errors in adolescents: is it reliable?. Archives Ophthalmol (Chicago Ill: 1960).

[14] Sankaridurg P, He X, Naduvilath T, Lv M, Ho A, Smith E, Erickson P, Zhu J, Zou H, Xu X (2017). Comparison of noncycloplegic and cycloplegic autorefraction in categorizing refractive error data in children. Acta Ophthalmol.

[15] Thorn F, Chen J, Li C, Jiang D, Chen W, Lin Y, Chang X, Deng R, Chen Y (2020). Refractive status and prevalence of myopia among Chinese primary school students. Clin Experimental Optometry.

[16] Wang J, Liu J, Ma W, Zhang Q, Li R, He X, Liu L (2021). Prevalence of myopia in 3-14-year-old Chinese children: a school-based cross-sectional study in Chengdu. BMC Ophthalmol.

[17] Matsumura S, Dannoue K, Kawakami M, Uemura K, Kameyama A, Takei A, Hori Y (2022). Prevalence of myopia and its Associated factors among Japanese Preschool Children. Front Public Health.

[18] Li T, Zhou X, Chen X, Qi H, Gao Q (2019). Refractive error in Chinese Preschool children: the Shanghai Study. Eye Contact Lens.

[19] Zhu B, Sun Y, Wang S, Qin X, Li L, Du B, Fu J, Wei R (2023). Refraction and ocular biometric parameters of preschool children in the Beijing whole childhood eye study: the first-year report. BMC Ophthalmol.

[20] Guo X, Fu M, Ding X, Morgan IG, Zeng Y, He M (2017). Significant Axial elongation with minimal change in refraction in 3- to 6-Year-old Chinese preschoolers: the Shenzhen Kindergarten Eye Study. Ophthalmology.

[21] Zhang L, He X, Qu X, You X, Wang B, Shi H, Tan H, Zou H, Zhu J. Refraction and Ocular Biometry of Preschool Children in Shanghai, China. *J Ophthalmol* 2018, 2018:5205946. 10.1155/2018/5205946 PMC5859869 29692930

[22] Wen G, Tarczy-Hornoch K, McKean-Cowdin R, Cotter SA, Borchert M, Lin J, Kim J, Varma R (2013). Prevalence of myopia, hyperopia, and astigmatism in non-hispanic white and Asian children: multi-ethnic pediatric eye disease study. Ophthalmology.

[23] Lam CS, Lam CH, Cheng SC, Chan LY (2012). Prevalence of myopia among Hong Kong Chinese schoolchildren: changes over two decades. Ophthalmic Physiological Optics: J Br Coll Ophthalmic Opticians (Optometrists).

[24] Lan W, Zhao F, Lin L, Li Z, Zeng J, Yang Z, Morgan IG (2013). Refractive errors in 3–6 year-old Chinese children: a very low prevalence of myopia?. PLoS ONE.

[25] Ma Y, Qu X, Zhu X, Xu X, Zhu J, Sankaridurg P, Lin S, Lu L, Zhao R, Wang L (2016). Age-specific prevalence of visual impairment and refractive error in children aged 3–10 years in Shanghai, China. Invest Ophthalmol Vis Sci.

[26] Huang L, Schmid KL, Yin XN, Zhang J, Wu J, Yang G, Ruan ZL, Jiang XQ, Wu CA, Chen WQ (2021). Combination effect of Outdoor activity and screen exposure on risk of Preschool Myopia: findings from Longhua Child Cohort Study. Front Public Health.

[27] Zadnik K, Sinnott LT, Cotter SA, Jones-Jordan LA, Kleinstein RN, Manny RE, Twelker JD, Mutti DO (2015). Prediction of juvenile-onset myopia. JAMA Ophthalmol.

[28] Benzir M, Afroze A, Zahan A, Naznin RA, Khanam A, Sumi SA, Haq MA, Lugova H, Haque M (2022). A study linking axial length, corneal curvature, and Eye Axis with demographic characteristics in the emmetropic eyes of Bangladeshi people. Cureus.

[29] Jin G, Liu Z, Wang L, Zhu Y, Luo L, Liu Y (2022). Corneal biometric features and their Association with Axial length in high myopia. Am J Ophthalmol.

[30] Han X, Xiong R, Jin L, Chen Q, Wang D, Chen S, Chen X, Ha J, Li Y, Qu Y (2022). Longitudinal changes in Lens Thickness and Lens Power among Persistent non-myopic and myopic children. Invest Ophthalmol Vis Sci.

[31] Li SM, Wei SF, Atchison DA, Kang MT, Liu LR, Li H, Li SY, Yang Z, Wang YP, Zhang FJ et al. Annual incidences and progressions of Myopia and High Myopia in Chinese Schoolchildren based on a 5-Year Cohort Study. Investig Ophthalmol Vis Sci 2022, 63(1). 10.1167/iovs.63.1.8 PMC8742535 34989760

[32] Hashemi H, Khabazkhoob M, Miraftab M, Emamian MH, Shariati M, Abdolahi-Nia T, Fotouhi A (2013). Axial length to corneal radius of curvature ratio and refractive errors. J Ophthalmic Vis Res.

[33] Zhao KK, Yang Y, Wang H, Li L, Wang ZY, Jiang F, Qu JF (2019). Axial length/corneal radius of curvature ratio and refractive development evaluation in 3-to 4-year-old children: the Shanghai Pudong Eye Study. Int J Ophthalmol.

[34] Guo XX, Fu M, Ding XH, Morgan IG, Zeng YF, He MG (2017). Significant Axial elongation with minimal change in refraction in 3-to 6-Year-old Chinese preschoolers the Shenzhen Kindergarten Eye Study. Ophthalmology.

[35] Lu YY, Jiang XM, Han X, Tan Q, Wu JS (2020). Proportion and characteristic of emmetropia in schoolchildren aged 6-11y: the Shenzhen elementary school eye study. Int J Ophthalmol.

[36] Huang D, Chen XJ, Gong Q, Yuan CQ, Ding H, Bai J, Zhu H, Fu ZJ, Yu RB, Liu H. Ocular biometric parameters among 3-year-old Chinese children: testability, distribution and association with anthropometric parameters. Sci Rep 2016, 6. 10.1038/srep29577 PMC4935861 27384307

[37] Li SM, Li SY, Kang MT, Zhou YH, Li H, Liu LR, Yang XY, Wang YP, Yang Z, Zhan SY (2015). Distribution of Ocular Biometry in 7-and 14-Year-old Chinese children. Optom Vis Sci.

[38] Liu S, Chen J, Wang J, Zhu Z, Zhang J, Zhang B, Yang J, Du L, Zhu J, Zou H et al. Cutoff values of axial length/corneal radius ratio for determining myopia vary with age among 3–18 years old children and adolescents. *Graefe’s archive for clinical and experimental ophthalmology = Albrecht von Graefes Archiv fur klinische und experimentelle Ophthalmologie* 2023. 10.1007/s00417-023-06176-0 37578514

[39] Yu X, Chen H, Savini G, Zheng Q, Song B, Tu R, Huang J, Wang Q (2018). Precision of a new ocular biometer in children and comparison with IOLMaster. Sci Rep.

[40] Zadnik K, Manny RE, Yu JA, Mitchell GL, Cotter SA, Quiralte JC, Shipp M, Friedman NE, Kleinstein R, Walker TW (2003). Ocular component data in schoolchildren as a function of age and gender. Optometry Vis Science: Official Publication Am Acad Optometry.

[41] Muralidharan G, Martínez-Enríquez E, Birkenfeld J, Velasco-Ocana M, Pérez-Merino P, Marcos S (2019). Morphological changes of human crystalline lens in myopia. Biomedical Opt Express.

[42] Chen Y, Wang D, Chen L, Yan W, He M (2021). Association of refraction and ocular biometry in highly myopic eyes. Clin Experimental Optometry.

[43] Rauscher FG, Francke M, Hiemisch A, Kiess W, Michael R (2021). Ocular biometry in children and adolescents from 4 to 17 years: a cross-sectional study in central Germany. Ophthalmic Physiological Optics: J Br Coll Ophthalmic Opticians (Optometrists).

[44] Khokhar S, Takkar B, Agarwal E, Gaur N, Ravani R, Venkatesh P (2018). Biometric evaluation of myopic eyes without posterior staphyloma: disproportionate ocular growth. Int Ophthalmol.

[45] Takkar B, Gaur N, Saluja G, Rathi A, Sharma B, Venkatesh P, Kumar A (2019). Evaluation of the vitreous chamber depth: an assessment of correlation with ocular biometrics. Indian J Ophthalmol.

[46] Kato K, Kondo M, Takeuchi M, Hirano K (2019). Refractive error and biometrics of anterior segment of eyes of healthy young university students in Japan. Sci Rep.

[47] Sng CC, Foo LL, Cheng CY, Allen JC, He M, Krishnaswamy G, Nongpiur ME, Friedman DS, Wong TY, Aung T (2012). Determinants of anterior chamber depth: the Singapore Chinese Eye Study. Ophthalmology.

[48] Tideman JW, Snabel MC, Tedja MS, van Rijn GA, Wong KT, Kuijpers RW, Vingerling JR, Hofman A, Buitendijk GH, Keunen JE (2016). Association of axial length with risk of uncorrectable visual impairment for europeans with myopia. JAMA Ophthalmol.

[49] AlMahmoud T, Priest D, Munger R, Jackson WB (2011). Correlation between refractive error, corneal power, and thickness in a large population with a wide range of ametropia. Invest Ophthalmol Vis Sci.

[50] Srivannaboon S (2002). Relationship between corneal thickness and level of myopia. J Med Association Thail = Chotmaihet Thangphaet.

[51] Chen MJ, Liu YT, Tsai CC, Chen YC, Chou CK, Lee SM (2009). Relationship between central corneal thickness, refractive error, corneal curvature, anterior chamber depth and axial length. J Chin Med Association: JCMA.

[52] Al-Mezaine HS, Al-Obeidan S, Kangave D, Sadaawy A, Wehaib TA, Al-Amro SA (2009). The relationship between central corneal thickness and degree of myopia among Saudi adults. Int Ophthalmol.

[53] Ortiz S, Mena L, Rio-San Cristobal A, Martin R (2014). Relationships between central and peripheral corneal thickness in different degrees of myopia. J Optometry.

[54] Zhou P, Wang DD, Fan L, Yang L, Zhao MW (2023). Thin central corneal thickness may be a risk factor for myopia progression in children. J Ophthalmol.

